# Exosomes derived from mesenchymal stem cells mediate miR-218 and inhibit proliferation, migration, and oxidative stress in retinal vascular endothelial cells via the EGFR/Akt/mTOR signaling pathway

**DOI:** 10.1097/MD.0000000000043989

**Published:** 2025-08-22

**Authors:** Wei Zhang, Yi Mu, Yichun Kong

**Affiliations:** a Tianjin Eye Hospital, Tianjin Key Lab of Ophthalmology and Visual Science, Tianjin Eye Institute, Clinical College of Ophthalmology, Tianjin Medical University, Tianjin, China; b Department of Ophthalmology, People’s Hospital of Hotan District, Xinjiang Uygur Autonomous Region, China; c Department of Ophthalmology, Tianjin Union Medical Center, Tianjin, P.R. China; d Tianjin NanKai Hospital, Tianjin Hospital of Integrated Chinese and Western Medicine, Tianjin Medical University NanKai Hospital, Integrated Chinese and Western Medicine Hospital, Tianjin University Nankai University Affiliated to Nankai Hospital, Tianjin, China.

**Keywords:** exosomes, miR-218, oxidative stress, retinal vascular endothelial cells, umbilical cord mesenchymal stem cells

## Abstract

Our study aims to elucidate the impact of exosomes obtained from human umbilical cord mesenchymal stem cells (MSC-Exos) on the proliferation, migration, and oxidative stress response of human retinal vascular endothelial cells (HRECs) and the mechanism. MSC-Exos were isolated from human umbilical cord mesenchymal stem cells using a low-temperature ultrahigh-speed centrifugation method. 3-(4,5-Dimethylthiazol-2-yl)-2,5-diphenyltetrazolium bromide assays, scratch tests, and cellular immunofluorescence assays were utilized to assess the influence of MSC-Exos on proliferation, migration, and oxidative stress in HRECs. Additionally, quantitative real-time PCR was utilized to quantify the expression levels of miR-218 in MSC-Exos and examine the underlying mechanisms involved in the regulation of oxidative stress. Electron microscopy and particle size analysis confirmed that the morphology of MSC-Exos was conformed to the morphological characteristics of exosomes and that MSC-Exos were positive for AlIX, CD63, and CD81 but negative for CytC. Reactive oxygen species levels were increased in HRECs induced by VEGF-165 and were decreased by MSC-Exo treatment. Compared with those of VEGF-165-transfected cells, the proliferation and migration of HRECs treated with MSC-Exos were greatly reduced, and the levels of tumor necrosis factor-a and IL-1β in the supernatant of HRECs were also significantly decreased (*P* < .05). The expression of miR-218 in HRECs treated with MSC-Exos was higher than that in the VEGF-165-transfected group (*P* < .05). The expression of VEGFA, VEGFB, HIF-1α, PGF, BDGF, and TGFβ1 in HRECs treated with MSC-Exos was significantly lower than that in the VEGF-165-transfected group (*P* < .05). After the overexpression of miR-218, the expression of EGFR, NLRP3, p-AKT/AKT, and p-mTOR/mTOR was significantly increased in HRECs (*P* < .05). MSC-Exos can significantly inhibit proliferation, migration and oxidative stress in HRECs by targeting miR-218 via the EGFR/Akt/mTOR signaling pathway.

## 1. Introduction

Diabetes is a chronic endocrine disease that damages many organs and tissues and is a global problem. At present, the number of diabetes patients in China is highest in the world, and the incidence rate is increasing annually.^[1]^ Human retinal vascular endothelial cells (HRECs) are unique vascular endothelial cells that directly transport glucose molecules to provide energy for retinal neurosensory layer cells. HRECs act as the inner barrier of the retina and are therefore highly susceptible to fluctuations in blood glucose levels, which in turn leads to pathological and functional changes.^[2]^ In patients with diabetes mellitus who have been exposed to high concentrations of glucose for a long time, high concentrations of glucose can induce HRECs to produce excessive oxygen free radicals, which can lead to oxidative stress damage, and the function of HRECs can be impaired, damaging the energy supply of the retinal nerve sensory layer and leading to a variety of retinopathies.^[3]^ Diabetic retinopathy (DR), a primary microvascular complication of both type 1 and type 2 diabetes, commonly results in diabetic macular edema, the leading cause of vision loss attributable to heightened retinal vascular permeability induced by elevated blood glucose levels.^[4]^ At present, DR has become a major blinding eye disease in adults worldwide and severely affects the daily life of patients.^[5]^ Despite the multitude of studies delving into the causes and disease mechanisms of DR, significant strides in its therapeutic options remain elusive, and further studies on innovative drugs and therapeutic targets are needed to improve the prognosis of patients with DR.

Human umbilical mesenchymal stem cell (hUMSCs) are stem cells that can self-renew and undergo multidirectional differentiation and play important roles in the regulation of inflammation and the repair of tissue damage. Studies have attempted to use hUMSCs in the treatment of DR.^[6]^ Exosomes, tiny membrane-bound vesicles measuring roughly 30 to 100 nm, are ubiquitously released by numerous cell types. These vesicles have a double-layered lipid membrane structure and can carry proteins, nucleic acids, lipids and other bioactive molecules to target cells and regulate a variety of physiological functions. Exosomes derived from hUMSCs are biocompatible and can be used as intercellular carriers to transport nucleic acids and proteins to recipient cells/target cells, which can affect the physiological and biochemical reactions of recipient cells/target cells. These factors can be used to treat a wide range of diseases.^[7]^ Notably, after being transplanted into humans, hUMSCs often undergo poor differentiation, increasing the risk of local tumorigenesis; the use of hUMSC-derived exosomes (human umbilical cord mesenchymal stem cells [MSC-Exo]) during transplantation can greatly reduce the risk of poor differentiation and neoplasia. Thus, MSC-Exos are for a less risky disease therapy than hUMSC-based therapeutic agents, and they have a wider range of therapeutic applications.^[8]^ Our previous research showed that miR-218-5p suppresses EGFR expression in pterygium tissues and human pterygium epithelial cells through the PI3K/Akt/mTOR signaling pathway.^[9]^ At present, we investigated the inhibitory effect of MSC-Exos on oxidative stress in HRECs induced by VEGF-165 and the molecular mechanism.

## 2. Materials and methods

### 2.1. Materials

This research was conducted with the approval of the Tianjin Eye Hospital’s Medical Ethics Committee(approval number: 2019035), adhering to the principles outlined in the Declaration of Helsinki. Human umbilical cord mesenchymal stem cells (hUCMSCs, C1181) and L929 mouse fibroblast cell line (BNCC100314), supplied by Tianjin Saier Biological Company (TEDA, Tianjin, China) were cultivated in 6-well plates for 48 hours. Dulbecco’s Modified Eagle Medium/Nutrient Mixture F-12 and fetal bovine serum (FBS) were obtained from GIBCO-Life Technologies (Grand Island). Endothelial cell medium was sourced from ScienCell Research Laboratories (Carlsbad). Mouse antihuman CD63, CD81, AlIX, CytC, VEGFA, VEGFB, HIF-1α, PGF, bFGF, EGFR, PI3K, AKT, p-AKT, mTOR, p-mTOR, NLRP3, and TGFβ1 antibodies, the isotype control antibody, and tumor necrosis factor (TNF)-α and IL-1β detection kit were obtained from Becton Dickinson Company (Franklin Lakes). The miR-218 mimics were purchased from Guangzhou and Rebo Biotechnology (Guangzhou, Guangdong, China).

### 2.2. Isolation, characterization, and measurement of hUCMSCs

Umbilical cords eligible for inclusion were rinsed 3 times in Petri dishes with 0.01 mmol/L phosphate-buffered saline (PBS), the umbilical cord was dissected, and the vein and perivascular umbilical cord tissue were isolated and set aside. Enzyme digestion was used to harvest hUCMSCs. One end of the umbilical vein was clamped with a hemostatic clamp, 2 g/L collagenase was injected into the umbilical vein, the other end was clamped, and the sample was incubated in a 5% CO_2_ incubator at 37°C for 20 minutes. The hemostatic forceps were opened, the digested fluid was poured into a petri dish. Afterward, 1 mL of FBS was introduced to halt the digestion process. The digested material was gathered into a 15 mL centrifuge tube and centrifuged at a moderate temperature (1000 rotations per minute) for 5 minutes. The liquid portion above the precipitated cells was discarded, and the remaining cells were resuspended in endothelial cell medium within the same centrifuge tube. Then, 20 mL of 2 g/L collagenase digestion solution was added, and the digestion was stopped when the precipitate changed from white to translucent. The cell suspension was completely covered with 200 mesh copper mesh. The mixture was centrifuged for 5 minutes at a speed of 1000 r/minute at room temperature, and subsequently supernatant was carefully removed. To the remaining cell pellet, Dulbecco’s Modified Eagle Medium/Nutrient Mixture F-12 medium mixed with 10% FBS was added, ensuring the cells received adequate nutrients. The culture medium was refreshed every 2 days to sustain cell health and promote growth. This in vitro culture process was carried out until the cells reached their third generation or passage (P3).

### 2.3. Isolation and characterization of exosomes

After 3rd to 5th generation hUCMSCs grew into a monolayer, 10 mL of serum-free medium was introduced, and following a 3-day incubation period, the conditioned medium was harvested and centrifuged at 10,000 r/minute for an hour to eliminate debris. The supernatant and 500 µL of exosome extraction reagent were mixed with 1 mL of cell medium, incubated at 4°C overnight, and centrifuged at 4°C and 14,000 r/minute for 60 minutes. The upper layer of the exosome-containing solution was removed and stored at −20°C.

The exosomes were fixed with glutaraldehyde at a concentration of 25 g/L, mixed with 10 g/L uranium acetate solution, and a drop was placed on a coated copper mesh, dried naturally and observed by transmission electron microscopy, after which images were taken. The number and size of the exosomes were measured using a nanoparticle tracking analyzer (Zetaview, Meerbusch, North Rhine-Westphalia, Germany). The solution of exosomes was diluted with PBS after analysis on the instrument, after which the relative concentration was determined according to the dilution coefficient. NTA 2.2 software (Meerbusch, North Rhine-Westphalia, Germany) was used for the data analysis. At least 4 separate counts were performed per sample, and the counts were averaged.

### 2.4. HREC culture and miR-218 and VEGF-165 overexpression

Human retinal endothelial cells (HRECs, C1265) were acquired from Tianjin Saier Biological Company (Tianjin, China). In vitro assays entailed the plating of HRECs at densities of 2 × 10^4^ cells/well in 96-well plates and 2 × 10^5^ cells/well in 24-well plates for further experimentation. The 3rd generation HRECs grown in wells was transfected by adding the vector at a multiplicity of infection and adding the infection-enhanced solution. HRECs were treated with 50 or 100 μg/mL MSC-Exos (RNA concentration). The cells were divided into the following groups: the VEGF-165-transfected HREC group, the miR-218-transfected HREC group, the negative control transfected HREC group, and the nontransfected HREC group. miRNA-218 mimics and a corresponding negative control were sourced from GenePharma (Shanghai, China). Transfections were conducted using Lipofectamine 2000 (Invitrogen, Carlsbad), following the manufacturer’s protocol, at a final concentration ranging from 50 to 100 nM. Post-transfection treatments were initiated after a 6-hour incubation period. HREC cells were transfected with VEGF-165 plasmids (4 μg for 6-well plates) using Lipofectamine 2000 reagent as described previously.

### 2.5. The viability of HRECs

The viability of HRECs was determined by 3-(4,5-dimethylthiazol-2-yl)-2,5-diphenyltetrazolium bromide (MTT) assays. In 96-well plates, HRECs across 4 groups were incubated with 20 μL of a 5 mg/mL MTT solution for a duration of 4 hours. Subsequently, the optical density at 490 nm was recorded automatically utilizing a multifunctional microplate reader, enabling the computation of cellular viability percentages.

### 2.6. HREC migration

The inoculated cells reached a confluence >90% were scratched vertically with a scratch tester. Following PBS washes, cells were maintained in serum-free medium for either 12 or 24 hours.

### 2.7. Reactive oxygen species (ROS)

To assess ROS generation, a fluorescent probe was employed. Cells seeded in 6-well plates were incubated with 10 μM 2',7'-dichlorofluorescin diacetate for 30 minutes, then rinsed twice with PBS. ROS visualization was accomplished through fluorescence microscopy examination. The DCF-DA probe can bind to ROS in cells and emit green fluorescence.

### 2.8. Cell culture supernatant

Enzyme-linked immunosorbent assay was used to measure the levels of TNF-a and IL-1b secreted by HRECs in each group. Standard wells were set up on an enzyme-labeled coated plate, and each group had 2 replicate wells. The results were automatically analyzed by measuring an enzyme marker, and the specific value was determined. The experiment was repeated 3 times, and the average value was taken as the final result.

### 2.9. Quantitative transcription PCR (qPCR)

Total RNA extraction was carried out using TRIzol reagent, with the extracted RNA preserved at −80°C for subsequent analyses. mRNA profiling involved cDNA synthesis utilizing the SUPERSCRIPTTM IV First-Strand Synthesis System (Takara, Otsu, Japan), followed by qPCR with SYBR Green PCR Master Mix (Solarbio, Beijing, China). miRNA analysis comprised cDNA synthesis with the Hairpin-itTM Real-Time PCR Kit (Genentech, Shanghai, China), employing U6 snRNA as the endogenous control with a standardized RT-qPCR kit from GenePharma (Shanghai, China). Glyceraldehyde-3-phosphate dehydrogenase served as the reference gene for mRNA normalization, while U6 was used for miRNA normalization. Expression fold changes were calculated using the 2^−ΔΔCT^ method.

### 2.10. Western blotting

The cells were collected, washed twice with cold PBS and centrifuged. The supernatant was combined with radioimmunoprecipitation assay buffer (containing protease and phosphatase inhibitors) and left to react on ice for 10 to 20 minutes with 10 to 15 seconds of shaking vibration to complete cell lysis. Post-centrifugation at 12,000 × g for 10 minutes at 4°C, the supernatant was carefully aliquoted into fresh tubes. Thereafter, protein concentrations were quantified using a bicinchoninic acid protein assay kit, and 6× sample-loading buffer and supernatant were mixed evenly. After the samples were heated at 95°C for 5 minutes, sodium dodecyl sulfate electrophoretic separation was carried out, and when the bromophenol blue stopped near the bottom of the plywood, the gel was removed for transfer (voltage 100 V for 50 minutes). Subsequently, the polyvinylidene difluoride membrane was separated, and blocking was performed with 2% bovine serum albumin for an hour. Incubation with primary antibodies ensued overnight at 4°C. Following this, the membrane was washed thrice with PBS containing Tween-20, and horseradish peroxidase-conjugated secondary antibody was applied for an additional hour.

### 2.11. Statistical analysis

Data analyses were conducted using SPSS version 13.0 software (Armonk). Results are presented as mean ± standard deviation (X ± s). Comparison between 2 groups was performed using an independent sample *t*-test. Statistical significance was set at *P* < .05.

## 3. Results

### 3.1. Isolation and characterization of exosomes

**Figure 1.**
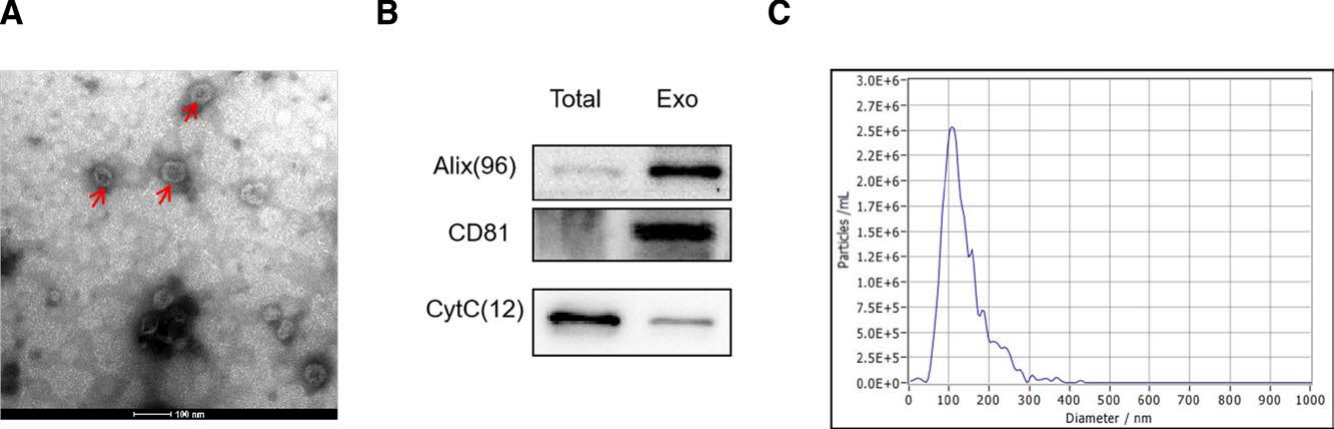
Characterization of exosomes. (A) The vesicles were round or oval in shape, and lipid-like membrane structures were observed around the vesicles (arrows). (B) Western blot analysis showed that the exosome membranes were rich in transmembrane 4 superfamily members (CD63, CD81, and Alix) and weakly expressed CytC. (C) The NanoSight analysis system demonstrated that MSC-Exos exhibited a predominant peak size of approximately 100 nm. n = 3 biological replicates. MSC-Exos = human umbilical cord mesenchymal stem cells.

Consistent with previous reports,^[4]^ hUCMSCs displayed strong positivity for CD73, CD90, and CD105, while exhibiting low expression levels of CD34, CD45, CD11b, CD19, and HLA-D. Transmission electron microscopy showed heterogeneous membrane vesicles with diameters of 40 to 100 nm. The vesicles were round or oval in shape, and lipid-like membrane structures were observed around the vesicles (Fig. 1A). Western blot analysis showed that the exosome membranes were rich in transmembrane 4 superfamily members (CD63, CD81, and Alix) and weakly expressed CytC (Fig. 1B). The NanoSight analysis system, through its advanced nanoparticle tracking technology, demonstrated that MSC-Exos exhibited a predominant peak size of approximately 100 nm (Fig. 1C).

### 3.2. Effects of MSC-Exos on oxidative stress in HRECs (ROS staining)

**Figure 2.**
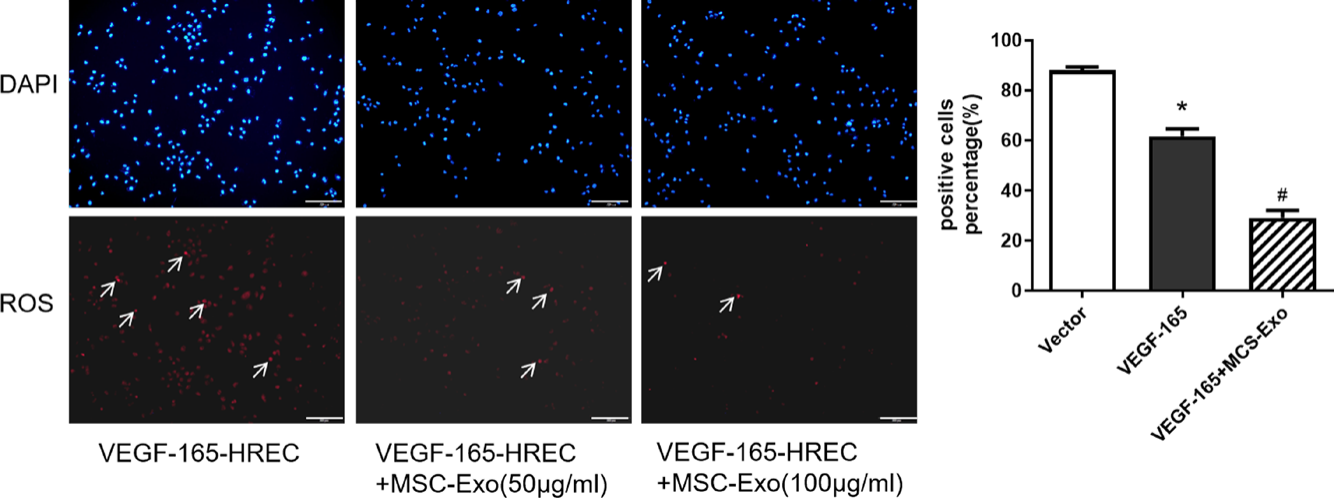
Effects of MSC-Exos on oxidative stress in HRECs. HRECs transfected with VEGF-165 exhibited obvious oxidative stress, and the level of oxidative stress was decreased after the addition of 50 or 100 μg/mL MSC-Exos, especially in response to 100 μg/mL. *P < .05 versus vector group; #*P* < .05 versus VEGF-165 group. Scale bar: 200 μm. n = 3 biological replicates. HRECs = human retinal vascular endothelial cells, MSC-Exos = human umbilical cord mesenchymal stem cells, ROS = reactive oxygen species.

HRECs were transfected with VEGF-165 to establish an oxidative stress model. The results showed that HRECs that were transfected with VEGF-165 exhibited obvious oxidative stress, and the level of oxidative stress dose-dependently decreased in response to different concentrations of MSC-Exos (Fig. 2).

### 3.3. The migration of HRECs was examined by a scratch test

**Figure 3.**
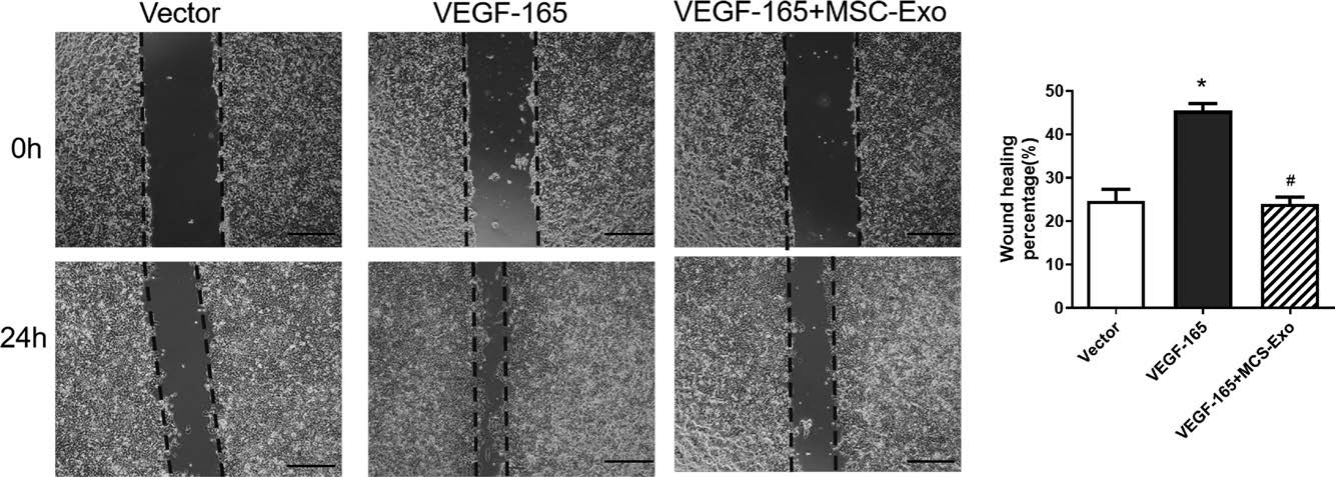
The migration of HRECs was examined by a scratch test. The percentage of migrating HRECs transfected with VEGF-165 was greatly up-regulated compared with those in the untreated control group, while the percentage of migrating HRECs was down-regulated in the MSC-Exo plus VEGF-165 group. *P < .05 versus vector group; #*P* < .05 versus VEGF-165 group. Scale bar: 100 μm. n = 3 biological replicates. HRECs = human retinal vascular endothelial cells, MSC-Exos = human umbilical cord mesenchymal stem cells.

The percentage of migrating HRECs transfected with VEGF-165 was greatly up-regulated compared with those in the untreated control group, while the percentage of migrating HRECs was down-regulated in the MSC-Exo plus VEGF-165 group. These findings showed that MSC-Exos could inhibit the migration of HRECs (Fig. 3).

### 3.4. The proliferation of HRECs was examined by MTT assays

**Figure 4.**
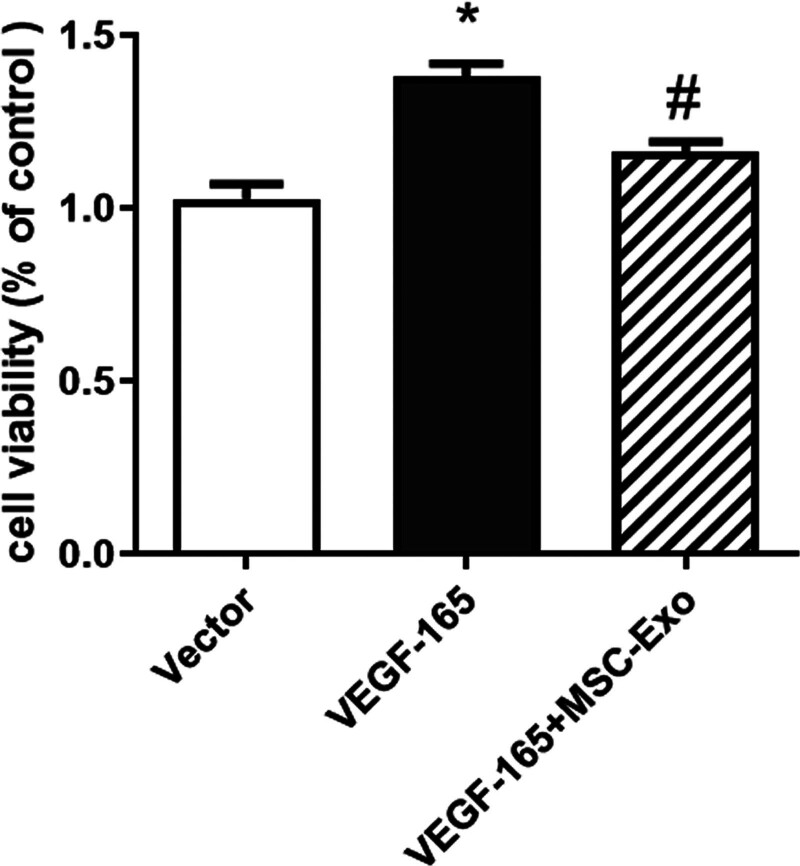
The proliferation of HRECs was examined by MTT assays. HREC proliferation was significantly increased in response to transfection with VEGF-165, and MSC-Exos inhibited HREC proliferation in response to transfection with VEGF-165. *P < .05 versus vector group; #*P* < .05 versus VEGF-165 group. n = 3 biological replicates. HRECs = human retinal vascular endothelial cells, MSC-Exos = human umbilical cord mesenchymal stem cells, MTT = 3-(4,5-dimethylthiazol-2-yl)-2,5-diphenyltetrazolium bromide.

The proliferation of HRECs was significantly elevated when the cells were transfected with VEGF-165 compared with that in the control group, and MSC-Exos inhibited the proliferation of HRECs transfected with VEGF-165 (Fig. 4).

### 3.5. The levels of TNF-a and IL-1b in the HREC culture supernatant were examined by enzyme-linked immunosorbent assay

**Figure 5.**
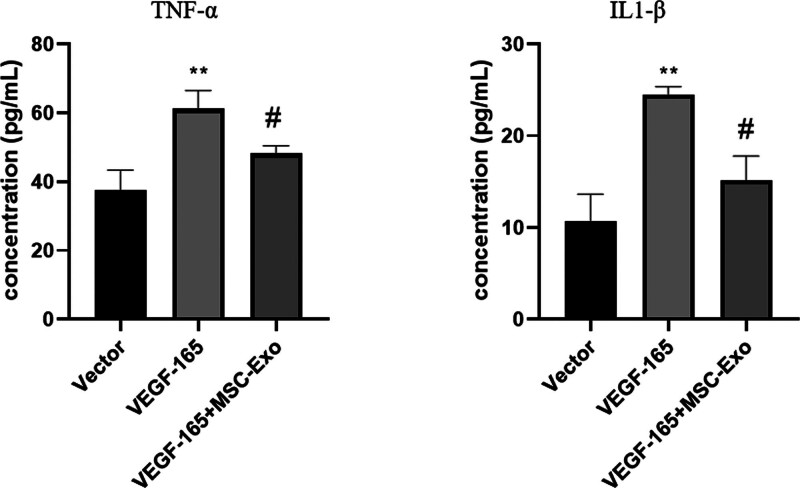
The levels of TNF-a and IL-1b in HREC culture supernatant were examined by ELISA. HRECs transfected with VEGF-165 exhibited increased secretion of inflammatory factors. A decrease in the secretion of TNF-a and IL-1b was observed after treatment with MSC-Exos.**P < .05 versus vector group; #*P* < .05 versus VEGF-165 group. n = 3 biological replicates. ELISA = enzyme-linked immunosorbent assay, HRECs = human retinal vascular endothelial cells, MSC-Exos = human umbilical cord mesenchymal stem cells, TNF = tumor necrosis factor.

HRECs transfected with VEGF-165 exhibited increased secretion of inflammatory factors. We found a decrease in the secretion of TNF-a and IL-1b after treatment with MSC-Exos (Fig. 5).

### 3.6. Impact of MSC-Exos on angiogenic factors and oxidative stress-related proteins in HRECs

**Figure 6.**
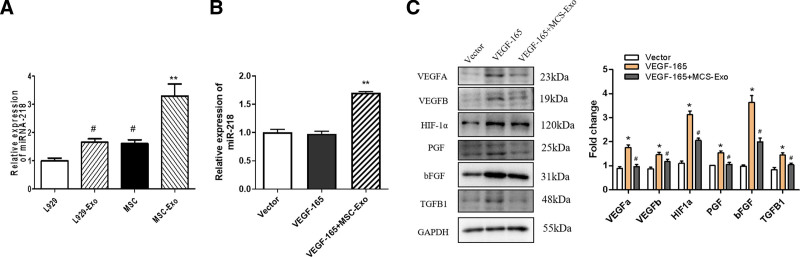
Effect of MSC-Exos on the expression of cell markers in HRECs. (A) The expression of miR-218 was found to be higher in MSC-Exos compared to both MSCs and L929-Exos. Conversely, no significant alteration in miR-218 levels was observed in either MSCs or L929-Exos. #*P* < .05 versus L929 group; **P < .05 versus L929-Exos group. (B) The expression of miR-218 in the MSC-Exo-treated group was increased, while the expression of miR-218 was not changed in the control group or in HRECs transfected with VEGF-165. **P < .05 versus vector group. (C) Western blotting confirmed that the protein levels of VEGFA, VEGFB, HIF-1α, PGF, bFGF, and TGFB1 were significantly increased in HRECs transfected with VEGF-165, while treatment with MSC-Exos decreased the expression of VEGFA, VEGFB, HIF-1α, PGF, bFGF, and TGFB1 in HRECs transfected with VEGF-165.*P < .05 versus vector group; #*P* < .05 versus VEGF-165 group. n = 3 biological replicates. HRECs = human retinal vascular endothelial cells, MSC-Exos = human umbilical cord mesenchymal stem cells.

The expression of miR-218 was found to be higher in MSC-Exos compared to both MSCs and L929-Exos. Conversely, no significant alteration in miR-218 levels was observed in either MSCs or L929-Exos (Fig. 6A). The level of miR-218 was elevated in the MSC-Exo-treated group, while the level of miR-218 was not changed in the control group or in HRECs transfected with VEGF-165 (Fig. 6B). Western blotting also showed that the expression of VEGFA, VEGFB, HIF-1α, PGF, bFGF, and TGFB1 were significantly increased in HRECs transfected with VEGF-165, while treatment with MSC-Exos decreased the expression of VEGFA, VEGFB, HIF-1α, PGF, bFGF, and TGFB1 in HRECs transfected with VEGF-165 (Fig. 6C).

### 3.7. Role of MiR-218 in modulating cell migration and signaling pathways in HRECs

**Figure 7.**
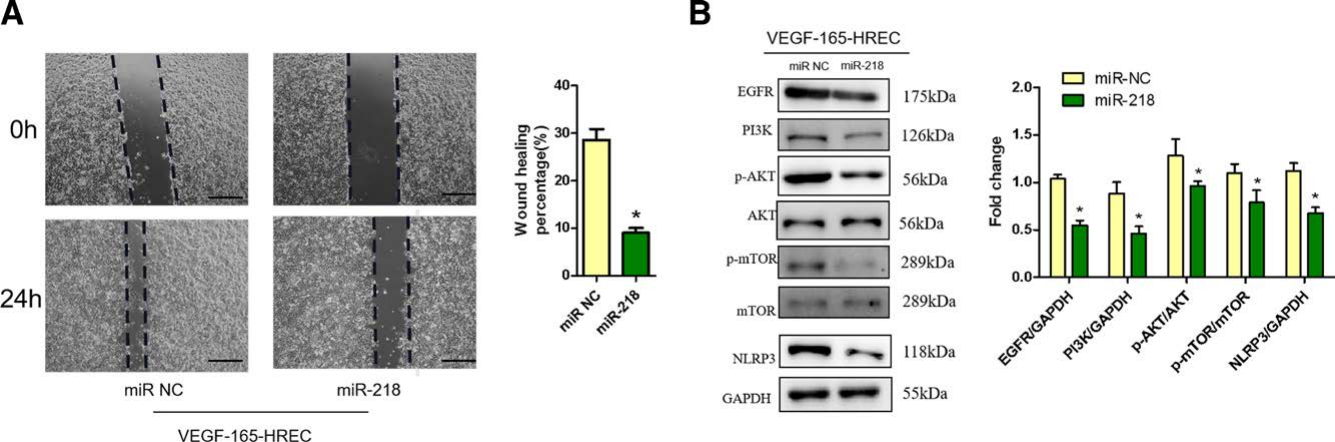
MiR-218 inhibited endothelial cell migration and the expression of cell markers. (A) The scratch test showed that miR-218 overexpression inhibited the migration of HRECs transfected with VEGF-165. (B) Western blot analysis indicated that miR-218 strongly decreased the protein levels of EGFR, PI3K, NLRP3, p-AKT/AKT, and p-mTOR/mTOR in vitro. *P < .05 versus miR-NC group. Scale bar: 100 μm. n = 3 biological replicates. HRECs = human retinal vascular endothelial cells.

The scratch test showed that miR-218 overexpression inhibited the migration of HRECs transfected with VEGF-165 (Fig. 7A). Moreover, Western blot analysis indicated that miR-218 strongly reduced the protein levels of EGFR, NLRP3, p-AKT/AKT, and p-mTOR/mTOR in HRECs transfected with VEGF-165 (Fig. 7B).

## 4. Discussion

Because the incidence and prevalence of DR are increasing worldwide, the need to develop effective therapeutic drugs and study their regulatory mechanisms is urgent. MSCs constitute a versatile class of stem cells capable of multi-directional differentiation. They are found in a variety of tissues, including bone marrow and fat.^[10,11]^ hUMSCs derived from the human umbilical cord have many advantages, such as anti-inflammatory and immunomodulatory effects, low immunoreactivity, ease of isolation in vitro and continuous subculture, and are a promising class of living cell tools for the treatment of various diseases.^[12,13]^ However, some studies have shown that hUMSCs can differentiate into cancer-associated fibroblasts after transplantation, which can enhance the growth and invasiveness of tumor cells and may even enhance the proliferation, carcinogenesis, and migratory capacity of cancer cells. In addition, hUMSCs can promote angiogenesis and induce antiapoptotic effects and drug resistance.^[14,15]^ MSC-derived exosomes do not possess the characteristics of their parent cells and can also reduce the risk of poor differentiation due to the transplantation of MSCs, which can lead to tumorigenesis; thus, these factors could be developed as treatments for disease. In addition, during in vitro culture of MSCs, exosomes are secreted in greater quantities than other factors and are easy to extract and store.^[16,17]^ Therefore, hUMSCs have additional advantages and were selected for exosome extraction. In this study, we successfully extracted exosomes from hUMSC culture supernatants and examined the morphology and volume of the extracted exosomes by NTA and electron microscopy, as described previously.^[18,19]^ The exosome markers CD63, CD9, and Alix were identified by Western blotting. It was confirmed that the exosomes were extracted successfully and that the components were reliable. At present, differential ultracentrifugation is the most commonly used method to extract exosomes and has the advantages of a large sample size and low extraction costs.

Exosomes were first identified as vesicle-like structures that bud during the differentiation of reticulocytes into mature erythrocytes.^[20,21]^ Exosomes from different cell sources reportedly carry conserved proteins and specific substances similar to those of the parent cells themselves; these proteins and substances are closely related to the physiological functions or pathological changes of the cells, and play important roles in tissue repair, injury, and the inhibition of inflammation.^[22,23]^ Further study has shown that exosomes regulate information exchange between cells; they play pivotal roles in both physiological and pathological aspects of the cardiovascular system, actively participating in the initiation and progression of cardiovascular disorders.^[24,25]^ Exosomes contain lipids, proteins, mRNAs, microRNAs and other components and can transmit genetic information to recipient cells through microRNAs and alter the modification and localization of regulatory proteins during transcription and translation, regulating signal-coupled pathways, enzymes, reactions, and self-regulation affect cellular biological function.^[26,27]^ However, the effect of exosomes on diabetic microvascular function has not been reported.

Several scholars have used MSC-exos to treat severe burn-induced hyperinflammation in vivo and in vitro, and MSC-exos were shown to suppress inflammatory responses by inhibiting the TLR4 and NF-κB signaling pathways, thereby alleviating excessive inflammation caused by severe burns.^[28,29]^ Another study showed that MSC-exos alleviated coronary artery disease by using protein supplementation to mitigate cardiac reperfusion injury.^[30]^ According to the results of our functional and mechanistic experiments, we found that MSC-exos could significantly decrease the activity of HRECs transfected with VEGF-165 and reduce the expression of VEGFA, VEGFB, HIF-1α, PGF, bFGF, and TGFB1. MSC-exos significantly decreased oxidative stress in HRECs and inhibited the production of TNF-a and IL-1b induced by VEGF-165. Our group confirmed that hUMSC-derived exosomes were enriched in miR-218 (Fig. 6A), validating that miR-218 was an important paracrine factor that helps hUMSCs mitigate HREC injury. The unique biological structure and function of MSC-exos could lead to the identification of a specific marker for early disease diagnosis and a targeted strategy for the treatment of microangiopathy in diabetes patients. Exosome-rich miR-218 protects against VEGF-induced damage in HRECs and inhibits cell proliferation and migration. MiR-218 also decreased the protein levels of EGFR, NLRP3, p-AKT/AKT, and p-mTOR/mTOR.

In China, with the improvements in living standards, the incidence of diabetes mellitus has increased, and diabetes mellitus is accompanied by retinal microangiopathy, which threatens the health of patients and eventually leads to blindness.^[31]^ This study was designed to investigate the protective effect of miR-218-enriched exosomes secreted by hUMSCs on VEGF-induced oxidative stress in HRECs to promote endothelial proliferation and migration and ameliorate endothelial cell dysfunction caused by VEGF. We provide reliable experimental evidence for the early treatment of DR. Our team is looking forward to providing experimental evidence for further improvements in microcirculation and gene therapy for DR, as well as further exploration of the role of MSC-exos in cell-to-cell signaling and regulation.

## Author contributions

**Conceptualization:** Wei Zhang.

**Data curation:** Yi Mu.

**Writing – review & editing:** Yichun Kong.
